# *RANK* hypomethylation is associated with primary osteoporosis in elderly men in Xinjiang: a case-control study

**DOI:** 10.1186/s12920-025-02282-6

**Published:** 2025-12-10

**Authors:** Zhuoya Maimaitiwusiman, Junjing Shang, Hong xiang, Xue Bai, Buluhan Halan, Hongmei Wang

**Affiliations:** The Second Department of Comprehensive, Internal Medicine of People’s Hospital of the Xinjiang Uygur Autonomous Region, Urumqi, 830001 China

**Keywords:** Osteoporosis, Methylation, *RANK* gene, Elderly community-dwelling adults, Case-control study

## Abstract

**Background:**

*RANK* is a candidate gene for osteoporosis based on both functional and genetic mechanisms. This study investigated the association between *RANK* methylation and osteoporosis among community-dwelling elderly people in Xinjiang, China.

**Methods:**

This case-control study was based on an epidemiological investigation. Initially, methylated cytosine-rich cytosine phosphate-guanine sites (CpGs) were identified within the *RANK* promoter region using a screening cohort. Subsequently, selected CpGs were detected via bisulfite sequencing in the primary cohort comprising 90 elderly men (47 with osteoporosis and 43 without). Finally, the correlations between osteoporosis and the methylation rates of the identified CpGs were assessed.

**Results:**

Age and prevalence of diabetes differed significantly between individuals with and without osteoporosis (*P* = 0.025 and *P* = 0.005, respectively), while other factors showed no statistically significant differences between the case and control groups (*P* > 0.05). The *RANK* methylation rate was significantly higher in the control group than in the osteoporosis group (*P* < 0.001). Furthermore, after covariance analysis to adjust for age, smoking, drinking, and diabetes, a significantly higher *RANK* methylation rate was observed in control individuals versus those with osteoporosis among elderly men from Xinjiang (*P* = 0.001). Multivariate logistic regression analysis, adjusted for confounding factors (age, smoking, alcohol consumption, and diabetes), further demonstrated that *RANK* hypomethylation was significantly associated with osteoporosis (odds ratio = 0.310, 95% confidence interval: 0.886–0.976).

**Conclusions:**

*RANK* hypomethylation shows a significant association with osteoporosis in elderly male residents in Xinjiang communities. These results support that *RANK* hypomethylation may play a role in the pathogenesis of osteoporosis.

## Background

Osteoporosis is a systemic metabolic bone disorder characterized by reduced bone mass, degradation of bone tissue structure, increased fragility, and heightened fracture risk. It affects one in three women and one in eight men over the age of 50 years [1]. In China, epidemiological data show that the prevalence of osteoporosis is 19.2% (6.0% in men, 31.2% in women, 16.2% in urban areas, and 20.7% in rural areas) in people over 50 years of age and 32.0% (10.7% in men, 51.6% in women, 25.6% in urban areas, and 35.3% in rural areas) in people over 65 years of age [2]. Consequently, osteoporosis and its associated fracture risk pose significant health challenges for aging populations [3]. Fractures may result from minor trauma such as falls or low-impact incidents. The condition also causes physical pain in elderly adults and imposes substantial economic burdens [4]. In China, the total cost of treating osteoporotic fractures reached $9.45 billion in 2010, with projections indicating a rise to $25.4 billion by 2050 [5].

The pathophysiology of osteoporosis remains incompletely understood, and many factors are known to increase the risk of osteoporosis, including sex, age, diet, exercise, and drug use [6]. Research has shown that epigenetic modifications play a vital role in gene expression regulation, encompassing DNA methylation, histone modification, and non-coding ribonucleic acid (ncRNA) [7, 8]. DNA methylation, the most common form of epigenetic modification, serves as a fundamental mechanism for genomic function regulation [9]. This process influences chromatin structure, DNA conformation, DNA stability, and protein-DNA interactions, thereby controlling gene expression. Methylation in eukaryotes usually occurs on cytosine-rich cytosine phosphate-guanine (CpG) islands in the gene promoter region. CpG islands represent critical regulatory sites for the formation and regulation of methylation in the human genome and are expected to be related to 60% of human gene promoters. Moreover, DNA methylation plays a vital role particularly in the pathogenesis of age-related diseases, including osteoporosis.

The receptor activator of the nuclear factor-κB (NF-κB) ligand (*RANKL*)/receptor activator of the *NF-KB* (*RANK*)/osteoprotegerin (*OPG*) pathway has been identified as the primary regulator of osteoporosis, and genetic variations in *RANK* have been linked to the pathogenesis of its osteoporosis [10]. Wang et al. (2018) demonstrated that DNA methylation decreases OPG transcriptional expression, which likely acts as a “main switch” in the pathogenesis of primary osteoporosis [11]. Cucumber seed peptide, a well-known traditional Chinese medicine, has been shown to treat osteoporosis by regulating the *RANK/RANKL/OPG* signaling pathway [12]. However, whether *RANK* methylation contributes to osteoporosis pathogenesis remains unclear. Therefore, this study aimed to investigate the association between *RANK* methylation and osteoporosis in elderly adults by sampling of the population in the Xinjiang communities.

## Materials and methods

### Study population

This study was approved by the Ethics Committee of the People’s Hospital of the Xinjiang Uygur Autonomous Region (Xinjiang, China). Written informed consent was obtained from all participants prior to data collection and biochemical testing.

A cross-sectional epidemiological survey of a general population was used for this case-control study, based on some data obtained for our previously published work [13]. Briefly, a three-stage stratified sampling strategy was used to select a representative sample of elderly adults (aged 60 years or older) residing in the Xinjiang communities. In the first stage, one county was randomly selected from both southern and northern Xinjiang (Mulei in northern Xinjiang and Luopu in southern Xinjiang). In the second stage, six townships were randomly selected within each county. In the third stage, five villages were randomly selected within each township. To ensure representative population samples, sample weights were calculated based on the sixth national census results of China.

The following inclusion criteria were applied for participation in the epidemiological survey: (1) age 60 years or older; (2) permanent resident; and (3) ability to walk independently without use of assistive devices. Exclusion criteria comprised: (1) severe cognitive impairment; (2) mental illness; (3) history of vital organ failure; (4) recent infection, acute cardiovascular disease, or recent surgery; (5) stroke sequelae; and (6) consumptive diseases such as malignant tumors or tuberculosis. The survey included 2100 eligible participants, with 1878 completing the study, yielding a response rate of 89.33%.

Participants completed a standardized questionnaire under the guidance of trained and certified physicians [13]. Blood pressure, weight, and height measurements were conducted by trained and qualified professionals using standardized protocols. Bone mineral density (BMD) assessments were performed according to the ACR Appropriateness Criteria Osteoporosis and Bone Mineral Density (2017), utilizing a portable bone mineral density analyzer (Osteosys EXA-3000, Korea) operated by certified technicians. Dual-energy X-ray absorptiometry was employed to measure BMD values at the forearm ulna and radius [14]. Individuals were asked to fast overnight prior to their study visit, and blood samples were drawn by venipuncture for measurement of serum biochemical indicators and the methylation rate of *RANK*. Blood samples were processed at the county hospital before being transported to Urumqi for storage at − 80 °C until analysis. Laboratory testing was conducted at the Clinical Laboratory Center of the People’s Hospital of the Uygur Autonomous Region of Xinjiang (Grade 3 A Hospital). Serum levels of total cholesterol, high- and low-density lipoprotein cholesterol, triglycerides, fasting blood glucose, 25-dihydroxyvitamin D3, folic acid, testosterone, creatinine, and serum calcium were measured by enzymatic methods.

### Research participants

**Fig. 1:**
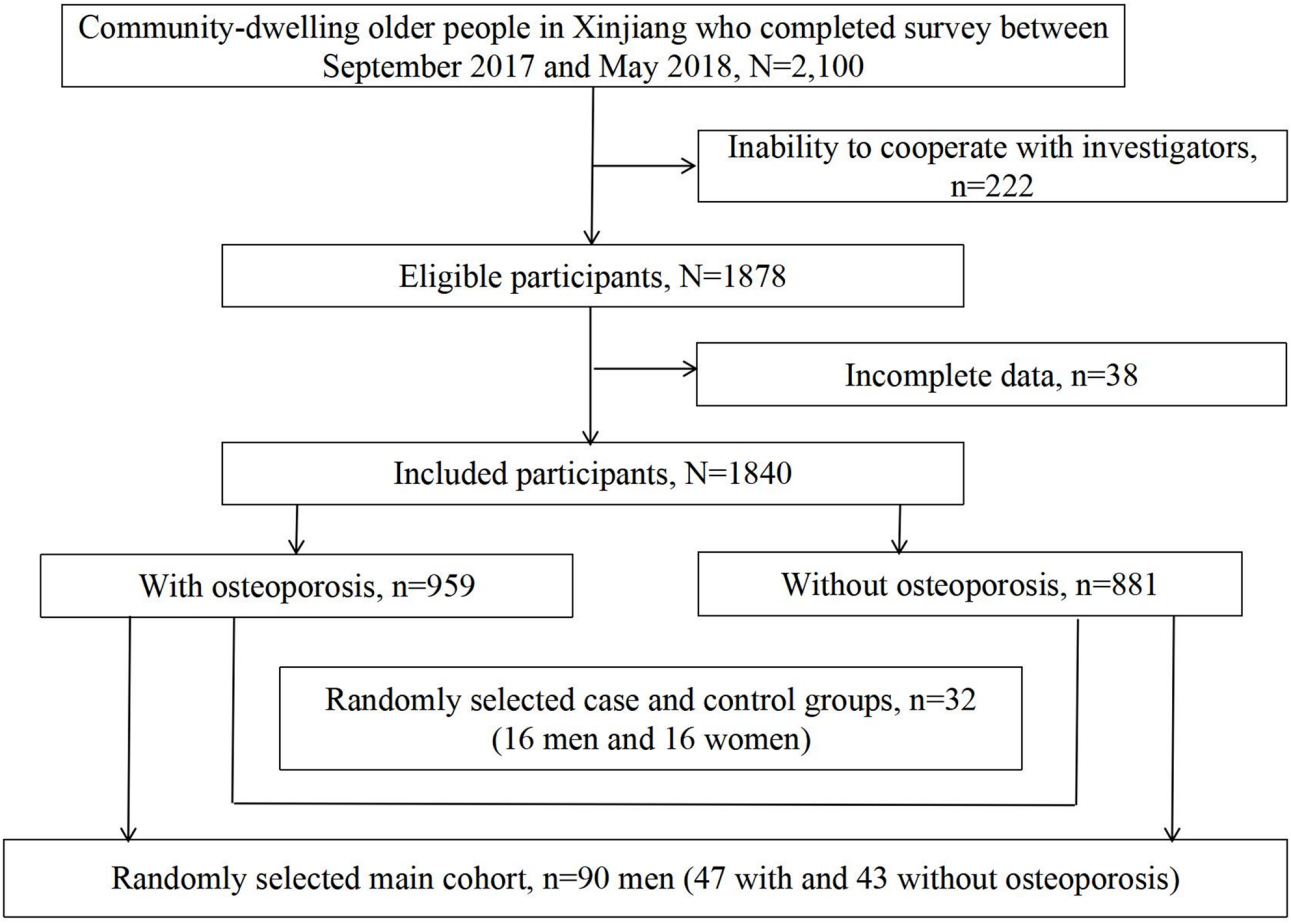
Selection procedure for the study participants

To identify the relevant methylated CpGs in the promoter region of *RANK*, where CpG sites are highly enriched, we initially selected a screening cohort of 32 individuals. This cohort included equal numbers from both the osteoporosis group (16 individuals) and without osteoporosis (16 individuals), with each subgroup comprising 8 males and 8 females to ensure equal representation. Since no significant representative CpGs were found in women, *we subsequently selected* 90 men (main cohort) from a database established through the aforementioned epidemiological survey. These participants were randomly assigned using computer-generated balanced block randomization into two groups: 47 individuals with osteoporosis and 43 without osteoporosis. Sample weights were determined according to the prevalence of osteoporosis in the study population. The selection procedure for the study participants is illustrated in Fig. 1.

### Diagnostic criteria and biochemical testing

Blood pressure measurements were obtained for each participant following a standardized protocol adapted from the American Heart Association recommendations. Trained observers measured body weight and height according to established procedures. Body mass index (BMI) was calculated using the following equation: BMI = body weight (kg)/[height (m)] [2]. Hypertension was defined by systolic blood pressure ≥ 140 mmHg and/or diastolic blood pressure ≥ 90 mmHg, or current use of antihypertensive medication. Diabetes mellitus was defined as an FBG level of ≥ 7.0 mmol/L or a previous diagnosis requiring treatment for type 2 diabetes mellitus requiring treatment. According to World Health Organization guidelines, smoking was defined as continuous smoking or smoking for more than 6 months, while drinking was defined as drinking at least once weekly with an intake of ≥ 8 g/week. For osteoporosis diagnosis in postmenopausal women and men aged ≥ 50 years, we adhered to criteria outlined in the “Guidelines for the Diagnosis and Treatment of Primary Osteoporosis (2017)” published by the Osteoporosis and Bone Mineral Salt Disease Branch of the Chinese Medical Association, which aligns with the World Health Organization (WHO) standards. Osteoporosis was diagnosed via dual-energy X-ray absorptiometry (DXA) when bone mineral density (BMD) was ≥ 2.5 standard deviations below the young adult mean.

### Detection of methylated CpGs in the RANK promoter region

Initially, we identified all CpG islands within the promoter region of *RANK* using bisulfite sequencing technology. This comprehensive sequencing of the *RANK* promoter region was conducted on blood samples from a carefully selected subgroup of the study cohort: 16 individuals diagnosed with osteoporosis, evenly divided by gender (8 males and 8 females), and 16 age- and gender-matched control participants without osteoporosis. Genomic DNA was extracted from the peripheral blood leukocytes of these participants utilizing the PAXgene Blood DNA kit (PreAnalytiX, Switzerland), ensuring high-quality DNA for subsequent analysis.

Genomic DNA was modified with sodium bisulfite using the EpiTect Fast Bisulfite Conversion Kit (QIAGEN, Germany). The modified DNA was then repeatedly eluted, purified, and stored at − 20 °C. All genomic DNA samples used for polymerase chain reaction (PCR) were deemed to be of good quality.

The PCR primers were designed using Methyl Primer Express v1.0 software. The sequence of the upstream primer was 5’-GGTGCCTTTCTAGAATTTGGTGG-3’, while the downstream primer sequence was 5’-CCAACCCTAAATTACCCTTCAC-3’. The resulting PCR product measured 376 bp in length and contained 20 CpG sites. The amplified PCR products were subsequently sent to Shanghai Biological Engineering Co., Ltd (Shanghai, China) for sequencing. The *RANK* sequencing results from patients and controls were compared using BiQ Analyzer analysis software.

### Detection of representative CpGs in elderly men in Xinjiang

**Fig. 2:**
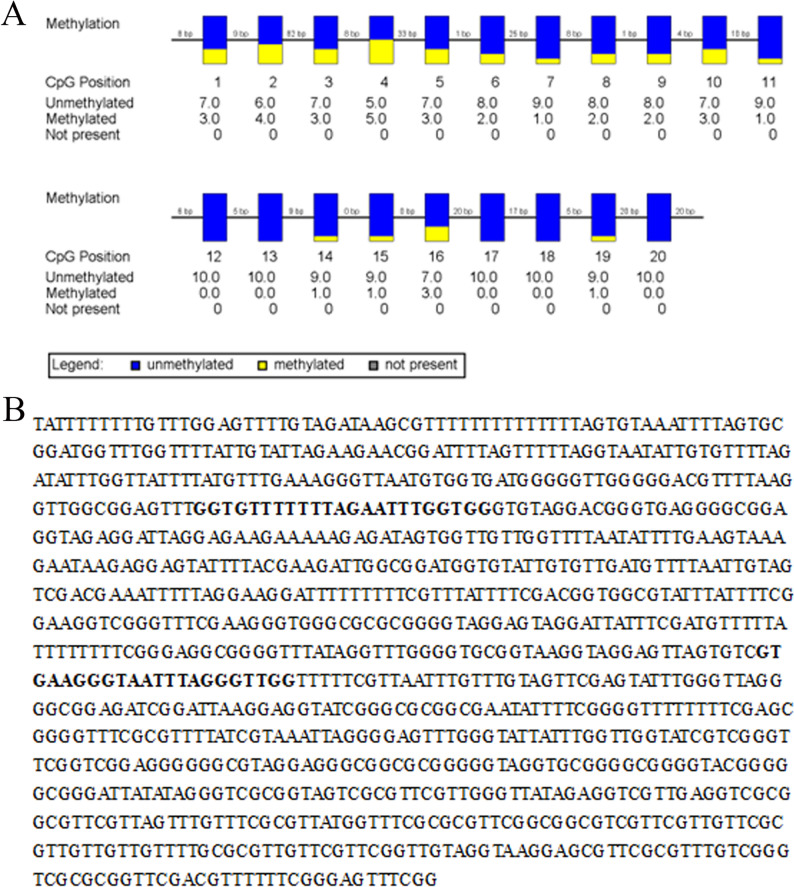
Analysis of CpG methylation sites in gene promoters. **A** Schematic diagram of RANK promoter CpG site. **B** Bisulfite sequencing results of the RANK promoter methylation profile

After assessing the relationships between osteoporosis and the methylation rate of the identified CpGs in male and female participants (Table 1), we analyzed 20 selected representative CpGs in 90 elderly men from the Xinjiang community using bisulfite sequencing (following the protocol described above) (Fig. 2). The study population comprised 47 osteoporotic men in the patient group and 43 men without osteoporosis in the control group.

**Table 1:** Screening for CpG Islands in RANK in the aged general population

Methylation rate (%)	Females			Males		
	Control group	Case group	*P*	Control group	Case group	*P*
	(*n*=8)	(*n*=8)		(*n*=8)	(*n*=8)	
CpG1	0.33 ± 0.191	0.21 ± 0.125	0.184	0.25 ± 0.12	0.28 ± 0.116	0.678
CpG2	0.68 ± 0.26	0.61 ± 0.21	0.606	0.54 ± 0.239	0.55 ± 0.12	0.897
CpG3	0.43 ± 0.198	0.38 ± 0.139	0.568	0.39 ± 0.173	0.33 ± 0.139	0.438
CpG4	0.48 ± 0.198	0.50 ± 0.001	0.727	0.49 ± 0.099	0.43 ± 0.089	0.205
CpG5	0.10 (0.00, ­0.10)	0.05 (0.00, ­0.10)	0.642	0.10 (0.10, ­0.25)	0.10 (0.03, ­0.18)	0.429
CpG6	0.10 (0.03, ­0.20)	0.10 (0.03, ­0.10)	0.513	0.20 (0.01, ­0.28)	0.15 (0.03, ­0.28)	0.662
CpG7	0.05 (0.00, ­0.10)	0.10 (0.00, ­0.18)	0.429	0.15 (0.10, ­0.28)	0.00 (0.00, ­0.00)	0.001*
CpG8	0.00 (0.00, ­0.00)	0.00 (0.00, ­0.00)	0.334	0.00 (0.00, ­0.18)	0.00 (0.00, ­0.00)	0.074
CpG9	0.10 (0.00, ­0.10)	0.10 (0.00, ­0.10)	0.693	0.20 (0.03, ­0.28)	0.00 (0.00, ­0.00)	0.002*
CpG10	0.00 (0.00, ­0.10)	0.10 (0.00, ­0.25)	0.245	0.20 (0.00, ­0.28)	0.05 (0.00, ­0.10)	0.065
CpG11	0.10 (0.00, ­0.18)	0.20 (0.10, ­0.20)	0.120	0.20 (0.10, ­0.28)	0.00 (0.00, ­0.08)	0.001*
CpG12	0.00 (0.00, ­0.00)	0.00 (0.00, ­0.08)	0.149	0.00 (0.00, ­0.08)	0.00 (0.00, ­0.00)	0.149
CpG13	0.00 (0.00, ­0.08)	0.05 (0.00, ­0.10)	0.334	0.00 (0.00, ­0.15)	0.00 (0.00, ­0.08)	0.506
CpG14	0.05 (0.00, ­0.18)	0.05 (0.00, ­0.18)	1.000	0.00 (0.00, ­0.08)	0.10 (0.03, ­0.10)	0.049*
CpG15	0.00 (0.00, ­0.08)	0.00 (0.00, ­0.00)	0.149	0.05 (0.00, ­0.10)	0.05 (0.00, ­0.10)	1.000
CpG16	0.00 (0.00, ­0.10)	0.00 (0.00, ­0.08)	0.619	0.15 (0.00, ­0.28)	0.05 (0.00, ­0.25)	0.575
CpG17	0.00 (0.00, ­0.00)	0.00 (0.00, ­0.08)	0.554	0.00 (0.00, ­0.00)	0.00 (0.00, ­0.00)	0.334
CpG18	0.00 (0.00, ­0.00)	0.00 (0.00, ­0.00)	0.334	0.00 (0.00, ­0.00)	0.00 (0.00, ­0.00)	0.334
CpG19	0.00 (0.00, ­0.10)	0.00 (0.00, ­0.08)	0.619	0.10 (0.00, ­0.18)	0.05 (0.00, ­0.10)	0.303
CpG20	0.05 (0.00, ­0.10)	0.05 (0.00, ­0.10)	1.000	0.00 (0.00, ­0.08)	0.00 (0.00, ­0.08)	1.000

*RANK methylation rate in the control group was significantly higher than that in the case group

### Statistical analysis

Data analysis were performed using Statistical Package for the Social Sciences software for Windows (version 26.0; SPSS Inc., Chicago, IL, USA). Values are presented as mean ± standard deviation (SD) or median (quartile), while categorical data are expressed as percentages. Patient characteristics were compared between the case and control groups using Student’s t-test or chi-square test. Differences in methylation rate distributions were analyzed using Student’s t-test for normally distributed data or Mann–Whitney U test for non-normally distributed data. Furthermore, analysis of covariance (ANCOVA) was conducted to assess and compare the distributions of methylation rates between the case and control cohorts, with adjustments for potential confounding variables including age, smoking, drinking, and diabetes. Finally, multivariate logistic regression analysis, adjusted for age, smoking, alcohol use, and diabetes, was conducted to evaluate the association between *RANK* methylation and osteoporosis.

## Results

### Methylation variations in CpGs of RANK in the screening cohort

**Fig. 3:**
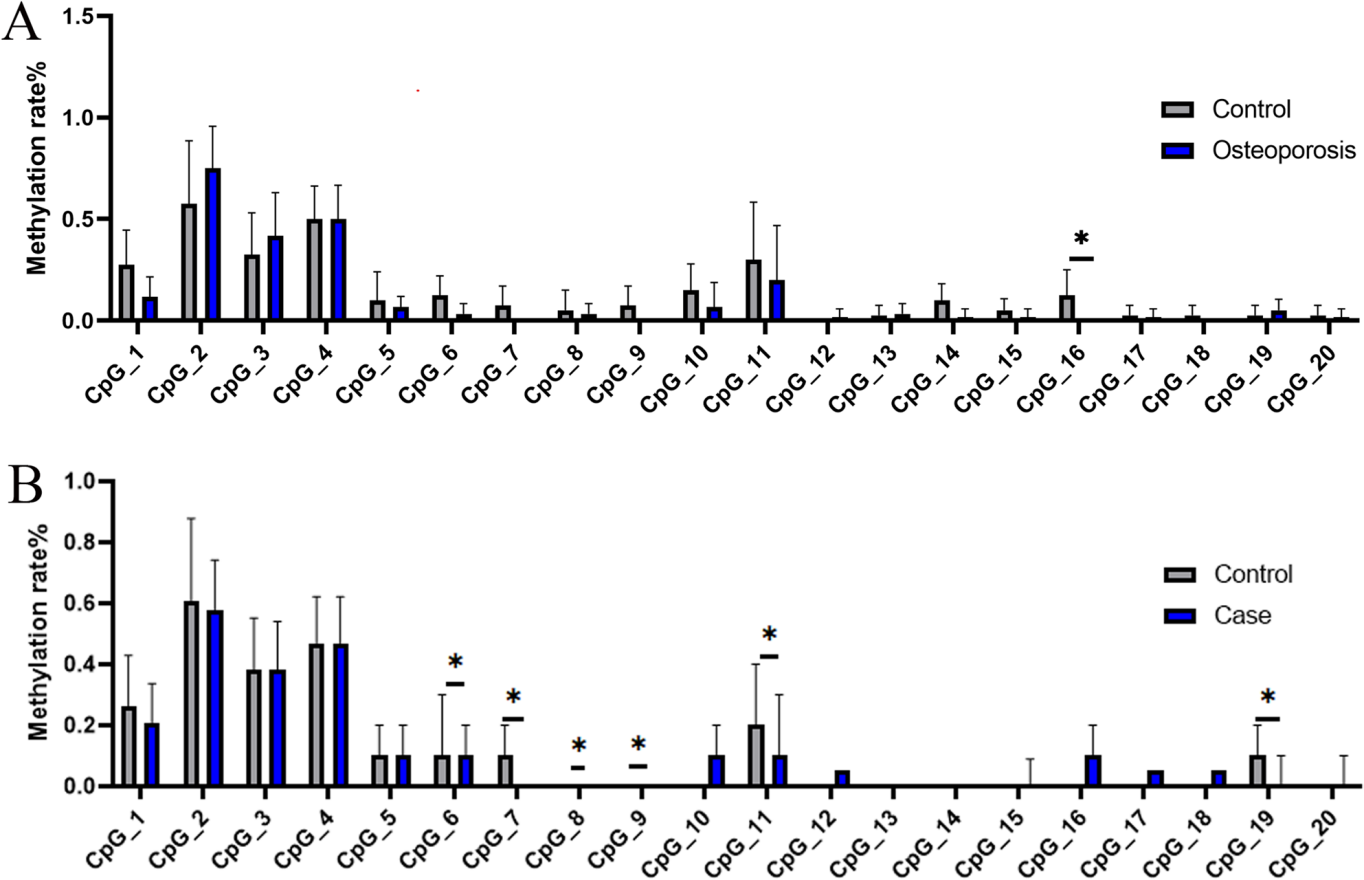
Methylation analysis of RANK CpG sites. **A** Boxplot showing methylation levels at RANK CpG sites in the screening cohort. **B** Boxplot of RANK CpG methylation in elderly men from the Xinjiang cohort

A total of 20 CpG islands were detected in the promoter region of *RANK* in the 32 individuals selected as the screening cohort. In males, four CpGs sites exhibited significantly higher methylation rates in the control group compared to the case group (CpG7, *P* = 0.001; CpG9, *P* = 0.002; CpG11, *P* = 0.001; CpG14, *P* = 0.049). In females, no statistically significant differences in methylation were observed for any of the 20 CpG sites between the case and control groups (Table 1; Fig. 3A).

Based on these results, the association between *RANK* methylation and osteoporosis was evaluated in the elderly male population living in the Xinjiang communities.

### Clinical characteristics of male participants

Table 2 presents the clinical characteristics of male participants included in the subsequent analysis. Significant differences were observed between case and control groups regarding age and diabetes prevalence (*P* = 0.025 and *P* = 0.005, respectively). No statistically significant differences were found between two groups for systolic and diastolic blood pressure, BMI, 25-dihydroxyvitamin D3 level, folic acid level, testosterone level, creatinine level, serum calcium concentration, and prevalence of smoking, drinking, and hypertension (*P* > 0.05).

**Table 2:** Clinical characteristics of male study participants

	Control group	Case group	t/χ^2^	*P*
	(*n* = 43)	(*n* = 47)		
Age (years)	68.53 ± 9.143	72.32 ± 6.484	2.246	0.025*
Systolic blood pressure (mmHg)	131.72 ± 8.719	130.17 ± 25.747	0.389	0.699
Diastolic blood pressure (mmHg)	81.30 ± 14.025	78.72 ± 10.498	0.980	0.330
BMI (kg/m^2^)	25.33 ± 3.838	24.43 ± 3.672	1.127	0.263
25-dihydroxy vitamin D3 (ng/mL)	15.63 ± 9.876	11.41 ± 8.179	1.785	0.078
Folic acid (ng/mL)	12.91 ± 6.088	14.51 ± 5.696	1.088	0.281
Testosterone (ng/mL)	3.83 ± 1.335	4.14 ± 1.107	0.928	0.358
Serum creatinine (µmol/L)	76.68 ± 18.456	76.44 ± 18.454	0.061	0.951
Serum calcium (mmol/L)	2.30 ± 0.136	2.31 ± 0.177	0.182	0.856
Smoking (No/Yes)	19/24	17/30	0.601	0.438
Drinking (No/Yes)	20/23	16/31	1.455	0.228
Hypertension (No/Yes)	15/28	19/28	0.293	0.588
Diabetes (No/Yes)	31/12	12/27	7.980	0.005*

*Age and prevalence of diabetes were significantly higher in the case group than in the control group

### Relationship between osteoporosis and RANK methylation rate in elderly men of the Xinjiang community

The *RANK* methylation rate was significantly higher in the control group than in the osteoporosis group (*P* < 0.001). Six CpG sites showed higher methylation rates in the control group compared to the case group (CpG6, *P* = 0.006; CpG7, *P* < 0.001; CpG8, *P* = 0.004; CpG9, *P* < 0.001; CpG11, *P* < 0.001; CpG19, *P* = 0.007, Table 3; Fig. 3B). Notably, all six differentially methylated CpG sites were localized within the RANK gene promoter region and exhibited a clustered distribution pattern in this genomic locus.

**Table 3:** Relationship between osteoporosis and RANK methylation rate in elderly men of Xinjiang

Methylation rate (%)	Control group	Case group	t(Z)	*P*
	(*n* = 43)	(*n* = 47)		
Total	51.74 ± 14.593	41.17 ± 9.042	4.088	< 0.001*
CpG1	0.26 ± 0.169	0.21 ± 0.127	1.536	0.128
CpG2	0.61 ± 0.269	0.58 ± 0.162	0.655	0.514
CpG3	0.38 ± 0.172	0.38 ± 0.160	0.015	0.988
CpG4	0.47 ± 0.152	0.47 ± 0.151	0.023	0.982
CpG5	0.10 (0.00 0.10)	0.10 (0.00 0.10)	1.695	0.090
CpG6	0.10 (0.00 0.20)	0.10 (0.00 0.10)	2.752	0.006*
CpG7	0.00 (0.00 0.10)	0.00 (0.00 0.00)	4.675	< 0.001*
CpG8	0.00 (0.00 0.10)	0.00 (0.00 0.00)	2.844	0.004*
CpG9	0.00 (0.00 0.10)	0.00 (0.00 0.00)	4.040	< 0.001*
CpG10	0.00 (0.00 0.20)	0.10 (0.00 0.10)	0.062	0.950
CpG11	0.20 (0.10 0.30)	0.10 (0.00 0.20)	3.720	< 0.001*
CpG12	0.00 (0.00 0.00)	0.00 (0.00 0.00)	0.519	0.604
CpG13	0.00 (0.00 0.00)	0.00 (0.00 0.10)	0.786	0.432
CpG14	0.00 (0.00 0.10)	0.00 (0.00 0.10)	0.503	0.615
CpG15	0.00 (0.00 0.10)	0.00 (0.00 0.09)	1.178	0.239
CpG16	0.00 (0.00 0.10)	0.00 (0.00 0.10)	1.269	0.204
CpG17	0.00 (0.00 0.00)	0.00 (0.00 0.00)	0.082	0.934
CpG18	0.00 (0.00 0.00)	0.00 (0.00 0.00)	0.953	0.340
CpG19	0.10 (0.00 0.10)	0.00 (0.00 0.10)	2.690	0.007*
CpG20	0.00 (0.00 0.10)	0.00 (0.00 0.10)	1.189	0.235

*RANK methylation rate was significantly higher in the control group than in the case group

Furthermore, after adjusting for age, smoking, drinking, and diabetes using ANCOVA, the *RANK* methylation rate was significantly higher in the control group than in the osteoporosis group among elderly men in the Xinjiang community (*P* = 0.001, Table 4).

**Table 4:** Adjusted RANK methylation rate between case and control groups in elderly Men, accounting for Age, Smoking, alcohol Consumption, and diabetes via ANCOVA

	Adjusted methylation rate (%)	Standard error	95% CI	F	*P*
Control group (*n* = 43)	50.48	1.718	47.067–53.895	11.019	0.001*
Case group (*n* = 47)	42.327	1.638	39.071–45.584		

### Association of RANK methylation with osteoporosis

After adjusting for confounding factors (age, smoking, drinking, and diabetes), multivariate logistic regression analysis also showed that reduced methylation rate of the *RANK* gene remained significantly associated with osteoporosis (OR = 0.930, 95% CI: 0.886–0.976; Table 5).

**Table 5:** Association of RANK methylation with osteoporosis in elderly men after adjustment for age, smoking, drinking, and diabetes

	OR	95% CI	*P*
Age	1.080	1.015–1.149	0.015
Smoking (Yes)	1.141	0.293–4.440	0.849
Drinking (Yes)	2.668	0.656–10.852	0.170
Diabetes (Yes)	1.908	0.583–6.247	0.285
Methylation rate (%)	0.930	0.886–0.976	0.003

## Discussion

Through systemic screening of CpG sites in *RANK* for methylation variations, this present study showed for the first time that a reduced *RANK* methylation rate was significantly associated with osteoporosis in a male cohort of community-dwelling elderly adults in Xinjiang. Six CpG sites exhibited significantly lower methylation rates in the case group compared to the control group.

Osteoporosis is a major chronic disease that diminishes the health of elderly adults. The pathogenesis of osteoporosis has been the subject of much research, which has shown that genetic factors play critical roles [15]. Gene methylation is considered a crucial component of the epigenetics involved in osteoporosis pathogenesis [16]. The onset of osteoporosis is closely related to bone loss caused by bone reconstruction imbalance. Several studies have reported that methylation of certain genes can affect the differentiation and cellular activity of osteoblasts and osteoclasts, as well as the occurrence and progression of osteoporosis [17, 18]. Delgado-Calle et al. (2013) investigated the femoral head region of patients with osteoporotic hip fracture and hip osteoarthritis and observed a significant difference in the methylation levels of 241 CpG loci and 228 genes between the two groups. Their findings indirectly suggested that osteoporosis may be associated with changes in the methylation levels of certain genes [19]. From their evaluation of whole blood cell RNA-sequencing and the level of DNA methylation in mesenchymal stem cells from young and elderly women, Roforth et al. reported different expression levels of promoter regions for 1528 sequences and differences in the methylation levels of some genes. These results suggest that the age-related decline in bone formation may be associated with methylation levels in mesenchymal stem cells and that osteoporosis caused by reduced bone formation may be associated, to some extent, with the methylation levels of certain genes [20].

The *RANK/RANKL/OPG* signaling pathway is known to play a critical role in the differentiation and activation of osteoclasts as well as their interaction with osteoblasts [21]. It has been reported that osteoclasts and osteoblasts engage in cell-cell contact through the RANKL/RANK/OPG pathway, which plays an important role in regulating bone remodeling. RANKL, a cytokine that promotes osteoclast formation and activation, binds to the RANK receptor on osteoclast precursor cells, inducing their differentiation and subsequent bone resorption [22]. Elevated RANKL expression and activity are associated with osteoporosis due to excessive bone resorption [22]. OPG, another cytokine, acts as a decoy receptor for RANKL, effectively blocking its interaction with RANK. This inhibition suppresses osteoclast differentiation and activation, thereby regulating bone resorption and maintaining bone density [23]. As a negative regulator of bone resorption, OPG contributes to bone homeostasis. An imbalance between RANKL and OPG—characterized by increased RANKL levels and decreased OPG levels—leads to excessive bone resorption and the development of osteoporosis [23]. *RANK* acts as a crucial regulatory factor in the *RANK/RANKL/OPG* signaling pathway, and a genome-wide association study confirmed an association between osteoporosis and methylation variations in *RANK* [24]. Emerging evidence indicates that DNA methylation in the *RANK* promoter region may directly influence osteoclastogenesis by modulating *RANK* expression [25]. Hypomethylation of *RANK* could increase its expression on osteoclast precursors, enhancing their sensitivity to *RANKL* signaling [26]. This activation triggers downstream transcription factors such as NF-κB and NFATc1, promoting osteoclast maturation and activity [27]. Previous studies have shown that LGR4 competes with *RANK* for *RANKL* binding, inhibiting classical *RANK* signaling during osteoclast differentiation [28, 29]. Additionally, Sun et al. found that methylation at specific sites in the *OPG*/*RANKL*/*RANK* pathway increases the risk of steroid-induced osteonecrosis of the femoral head [30]. Wang et al. reported abnormal methylation at multiple CpG sites in the *OPG*/*RANKL*/*RANK* genes of peripheral blood leukocytes in Chinese men with alcohol-induced osteonecrosis [31]. These findings suggest that RANK gene methylation levels may play a crucial role in osteoporosis pathogenesis and progression in elderly adults. In our study, we observed significantly higher *RANK* gene methylation rates in the control group compared to the osteoporosis group among elderly men in Xinjiang communities. However, in the screening cohort, no significant methylation differences were detected in females, possibly due to limited statistical power from the small sample size (only 8 female cases and 8 controls in the initial CpG screening analysis). While biological sex differences in *RANK* methylation cannot be entirely excluded, the modest sample size likely restricted our ability to identify meaningful associations in females. Future studies with larger female cohorts are needed to explore potential sex-specific epigenetic mechanisms in osteoporosis development.

Notably, Xinjiang’s unique geographical environment and ethnic composition may potentially influence the study outcomes. The region’s northwestern location provides abundant sunlight, which could affect vitamin D synthesis [32]. However, this study found no statistically significant difference in 25- hydroxyvitamin D3 levels between case and control groups (*P* = 0.078), suggesting that vitamin D may not be the primary driver of methylation differences in this population. Ethnic and regional variations in diet and lifestyle may further modulate these pathways. For instance, the Xinjiang population, particularly pastoral communities, traditionally consumes a diet rich in dairy products and animal proteins, which could influence bone metabolism through calcium intake and acid-base balance [33]. While our analysis focused on a homogeneous cohort of Xinjiang Uyghur elderly males, the findings may not be generalizable to other populations. Substantial inter-population heterogeneity could exist in vitamin D metabolism, dietary profiles, and epigenetic signatures due to variations in solar exposure gradients (e.g., southern China vs. northwest China), cultural practices, and genetic ancestry. Our results demonstrate that *RANK* promoter hypomethylation is significantly associated with osteoporosis in this Xinjiang-based cohort of community-dwelling elderly men (adjusted for age, smoking status, alcohol consumption, and diabetes mellitus). Furthermore, differential methylation analysis revealed hypomethylation at six specific CpG sites in osteoporotic patients compared to controls. These findings underscore the necessity for future multi-ethnic, geographically stratified studies to elucidate the interaction between environmental modifiers, genetic background, and epigenetic mechanisms in osteoporosis pathogenesis.

Xinjiang is located in northwest China, and residence in the pastoral areas of Xinjiang is relatively fixed. Environmental factors affecting bone mass, such as habitat, eating habits, lifestyle, and occupation, are relatively consistent [34]. Therefore, this community-dwelling cohort provides an ideal population for studying the genetic mechanisms of complex diseases. Furthermore, this study was based on an epidemiological investigation from which participants were randomly selected. Consequently, the results of this study are highly reliable. Despite these strengths though, this study has some limitations. First, participants were only Chinese individuals from a geographically restricted area; further studies are needed to determine generalizability to other populations. Second, although our key finding of RANK hypomethylation associated with osteoporosis in elderly men used rigorous randomization, the modest sample size (*n* = 90) may limit statistical power. Future large-scale prospective cohorts are required to confirm these epigenetic associations and uncover novel mechanistic insights. Third, this investigation did not establish causality between RANK hypomethylation and downstream molecular effects, particularly alterations in RANK expression or dysregulation of the RANK/RANKL/OPG axis. Targeted mechanistic experiments are underway to address this gap in osteoimmunology. Fourth, due to the limited sample size and significant group differences in age and diabetes prevalence, stratified analyses by age or diabetes status were infeasible. Although we adjusted for age, smoking, drinking, and diabetes in ANCOVA and multivariate regression, future large-scale prospective studies are needed to confirm findings and investigate subgroup-specific effects. Furthermore, due to the exploratory nature of this study, we did not examine whether the six differentially methylated CpGs (CpG6, 7, 8, 9, 11, 19) overlap with key regulatory elements (e.g., transcription start sites or transcription factor binding motifs) or analyze their co-methylation patterns. These questions will be prioritized in future functional experiments, including promoter activity assays and epigenetic mechanistic validation.

## Conclusions

In summary, hypomethylation of *RANK* was found to be significantly associated with osteoporosis in community-dwelling elderly men in Xinjiang, suggesting that *RANK* hypomethylation may be involved in the pathogenesis of osteoporosis.

## Data Availability

The data sets used and/or analyzed during the current study are available from the corresponding author upon reasonable request.

## Abbreviations

CpGs: Cytosine-phosphate-Guanine
DNA: Deoxyribonucleic acid
ncRNA: Non-coding ribonucleic acid
NF-κB: Nuclelar factor-κB
RANK: receptor activator of nuclear factor kappa-B
RANKL: receptor activator of nuclear factor kappa-B ligand
OPG: Osteoprotegerin
BMD: Bone mineral density
BMI: Body mass index
WHO: World Health Organization
DXA: Dual-energy X-ray absorptiometry
PCR: Polymerase chain reaction
